# Update on MRI in pediatric intracranial germ cell tumors—The clinical and radiological features

**DOI:** 10.3389/fped.2023.1141397

**Published:** 2023-05-04

**Authors:** Mingwen Yang, Jian Wang, Lin Zhang, Jungang Liu

**Affiliations:** Department of Radiology, Xiamen Children’s Hospital, Children’s Hospital of Fudan University at Xiamen, Xiamen, China

**Keywords:** germ cell tumor, brain tumor, germinoma, NGGCTs, neuroimaging, magnetic resonance imaging

## Abstract

Intracranial germ cell tumors (iGCTs) are uncommon brain tumors that mainly occur in children. Differing in histology, location, and gender of the patients, iGCTs are often divided into germinomas and non-germinomatous germ cell tumors (NGGCTs). Early diagnosis and timely treatment are crucial to iGCTs, the subtypes of which have substantial variations. This review summarized the clinical and radiological features of iGCTs at different sites, and reviewed the recent advances in neuroimaging of iGCTs, which can help predict tumor subtypes early and guide clinical decision-making.

## Introduction

Intracranial germ cell tumors (iGCTs) are uncommon brain tumors that account for approximately 0.9% of all pediatric tumors, 3.7% of pediatric brain tumors, and 28.7% of germ cell tumors overall (1–3). iGCTs are more frequently diagnosed in East Asian countries (4, 5), and the incidence is much lower in North American and European countries (4, 6). These tumors are more common in children and adolescents; the peak age of diagnosis is between 10 and 19 years old, and about 71% are diagnosed before 20 years old (7). The central nervous system (CNS) is the second most common site of extragonadal GCTs, following the mediastinum (5, 8).

According to the classification of tumors by the World Health Organization (WHO) in 2021, iGCTs can be divided into germinoma, choriocarcinoma, yolk sac tumor, embryonal carcinoma, teratoma (immature and mature), teratoma with somatic-type malignancy, and mixed germ cell tumor (9). According to pathology and laboratory characteristics (including tumor markers), iGCTs are often divided into germinomas and non-germinomatous germ cell tumors (NGGCTs). Germinomas account for approximately 50%–70% of cases and NGGCTs account for the remaining cases (6, 10).

Intracranial GCTs mainly occur in the midline supratentorial, such as the pineal region (50%) and suprasellar (20%–30%); about 6%–10% of tumors occur in off-midline intracranial structures, including the basal ganglia and thalamus, and rarely in the cerebellum (11). In 6%–13% of cases, synchronous tumors are found in the pineal and suprasellar regions, termed a “bifocal germinoma” (12).

Neuroimaging provides important value for the diagnosis, surgical or medical planning, assessment of treatment response, and follow-up of iGCTs. However, there is considerable overlap in the imaging features of iGCTs, so it is necessary to improve the accuracy of preoperative differential diagnosis by combining the clinical information and tumor markers. Therefore, this review aims to summarize the clinical and radiological features of iGCTs and review the recent advances in neuroimaging of iGCTs.

## Characteristics of germinomas at different sites

### Pineal region

Germinomas are the most common primary tumor in the pineal region, and the incidence rate of males is significantly higher than that of females (13, 14). Clinical symptoms are related to the mass effect on the adjacent structures and invasion of surrounding structures, such as obstructive hydrocephalus, the Parinaud syndrome, and other symptoms of endocrine disorders (12).

**Figure 1:**
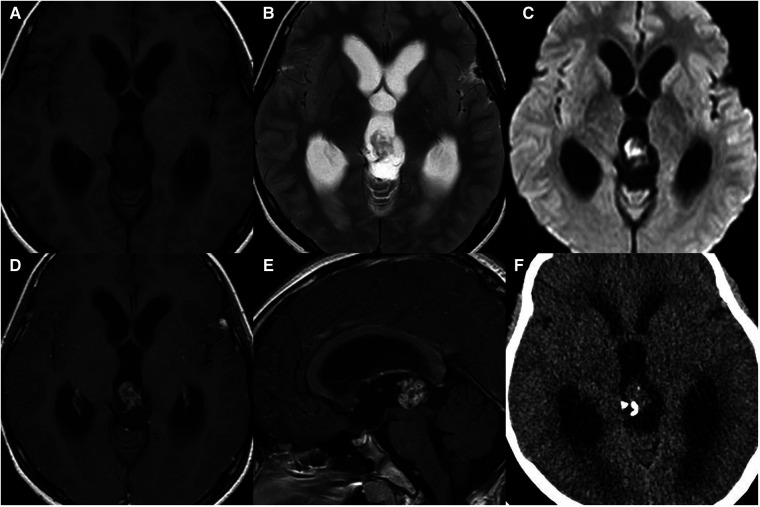
11-year-old boy with a pineal germinoma: axial T1WI (**A**), T2WI (**B**), and DWI (**C**), axial and sagittal post-contrast T1WI (**D,E**), and axial unenhanced CT (**F**) show a pineal mass with cystic change and engulfed calcification.

**Figure 2:**
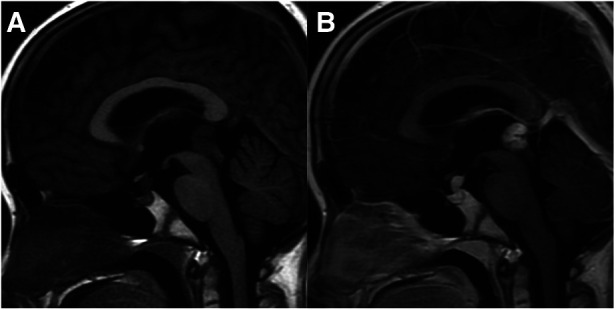
10-year-old boy with bifocal germinomas: sagittal T1WI (**A**) and post-contrast T1WI (**B**) show bifocal germinomas both in the suprasellar and pineal region.

The typical imaging appearances are nodular or lumpy masses in the pineal region, usually with clear boundaries and significantly homogeneous enhancement. The tumors are typically hyperdense with “engulfed” calcification on CT, but the calcification is usually not observed until at least 4 years old. They usually are isointense to hyperintense on T2WI, and hypointense to isointense on T1WI. Cystic changes may be observed while hemorrhage and necrosis are rare (Figure 1). The parenchymal portion of the tumor is usually isointense or slightly hyperintense on T2* gradient echo/susceptibility weighted imaging (T2* GRE/SWI), the internal intensity is relatively homogeneous, and if it is significantly heterogeneous, which would be made up of hemorrhage or cystic or fat, it is more likely a NGGCT (11). In addition, reduced apparent diffusion coefficient (ADC) values, bithalamic extension, and thick peritumoral edema are significant features that are more frequent in germinomas than in NGGCTs (4, 15). A recent study demonstrated lower relative cerebral blood flow and time-to-peak values which were obtained by dynamic susceptibility contrast perfusion weighted imaging (DSC-PWI) (16). Elevated lipid peaks and taurine peaks maybe observed on 1H-magnetic resonance spectroscopy (1H-MRS), although these features are not specific and not always present (17, 18). The tumors can be planted and spread along the cerebrospinal fluid (CSF); bifocal tumors both in the pineal region and suprasellar region strongly suggest the diagnosis of germinomas (Figure 2), however, NGGCTs cannot be excluded (19). If pineal germinoma is suspected, the MRI of the entire neuroaxis should be imaged (18). Moreover, demographical-radiomics models are also helpful for distinguishing germinoma from other tumors in the pineal region, with features extracted from demographic information, tumor, edema and MRI sequences (20).

**Figure 3:**
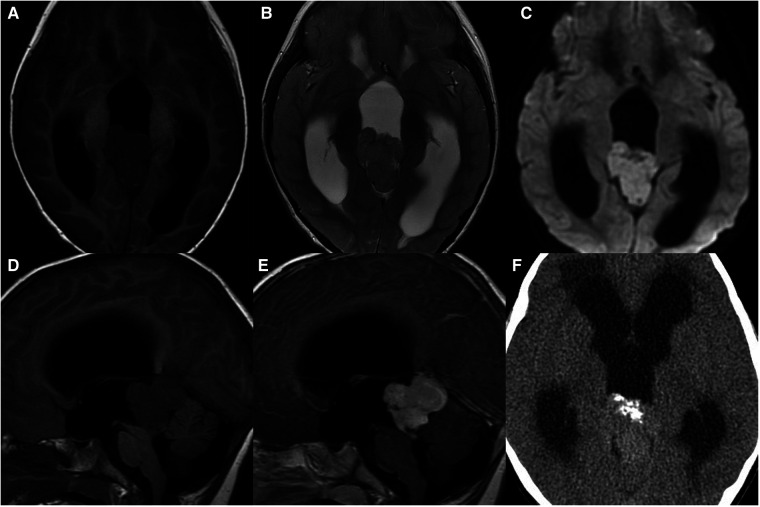
4-year-old boy with a pineoblastoma: axial T1WI (**A**), T2WI (**B**), and DWI (**C**), sagittal T1WI and post-contrast T1WI (**D,E**) show a lobulated mass with restricted diffusion, enhancement, and hydrocephalus; axial unenhanced CT (**F**) shows exploded calcification.

Differential diagnosis: (1) NGGCTs: See NGGCTs Part in detail; (2) Pineoblastoma: Pineoblastomas are highly malignant tumors arising directly from pineocytes, which are most frequently discovered in infancy and childhood, with a median age at diagnosis of 4.9 years old, which is lower than germinomas (21, 22). On CT, pineoblastomas are large and hyperdense with peripheral or “exploded” calcification (Figure 3). These tumors are frequently heterogeneous, hypointense to isointense on T1WI and isointense to hyperintense on T2WI, with restricted diffusion and variable enhancement. Internal hemorrhagic and cystic components may also be seen. It is prone for the tumors to invade adjacent structures and spread along CSF, and these tumors are the most frequent cause of hydrocephalus. It is rare for pineoblastoma to occur simultaneously with retinoblastoma, a configuration termed “trilateral retinoblastoma”. (3) Pineocytoma: Pineocytomas are benign tumors originating from the pineal gland. They are often encountered in middle-aged adults, with no significant gender difference (23). The tumors are slightly hyperdense on CT with clear boundaries and peripheral calcification, and slightly hypointense on T1WI and hyperintense on T2WI, without evidence of restricted diffusion. Hemorrhage, necrosis, and cystic change are rare. No or mild to moderate enhancement can be observed. Implant metastasis is rare.

### Suprasellar region

Suprasellar region germinomas are more common in children and slightly more common in girls than in boys (24, 25). Symptoms often related to endocrine disorders, such as diabetes insipidus, visual acuity decline, visual field defect, and hypopituitarism of different degrees, may also occur (6). Diabetes insipidus is the most common presentation for suprasellar germinomas.

**Figure 4:**
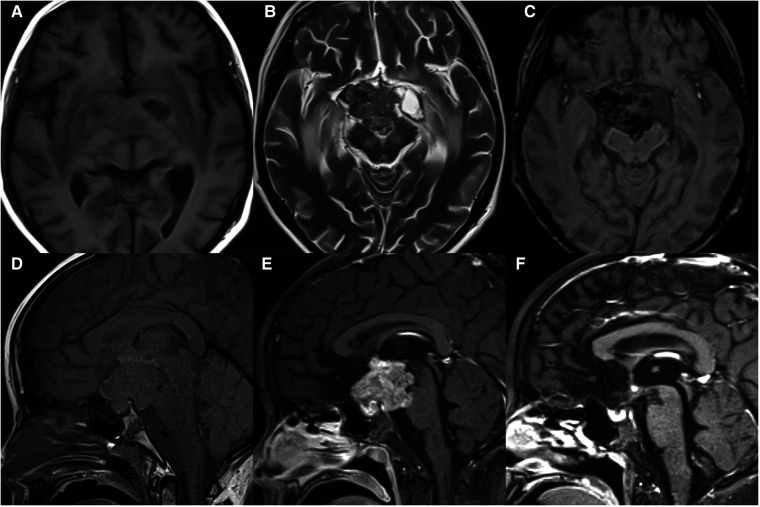
11-year-old girl with a suprasellar germinoma: axial T1WI (**A**), T2WI (**B**), and SWI (**C**), sagittal T1WI (**D**) and post-contrast T1WI (**E**) show an ill-defined irregular mass with necrosis, cysts, and hemorrhage, involving optic chiasma and pons; the normal high signal in the posterior pituitary disappears; sagittal post-contrast T1WI (**F**) shows significantly reduced mass after radiotherapy.

On MRI, typically the tumors originate from the floor of the third ventricle (hypothalamus) or pituitary infundibulum and can extend into the sella and involve the posterior pituitary. The tumors are mainly solid, with clear boundaries, and can compress adjacent structures. The tumors are commonly ill-defined irregular masses with necrosis, cysts, and hemorrhage, but without calcification. They have been demonstrated to be hypointense to isointense on T1WI, isointense to hyperintense on T2WI, and hyperdense on CT, with markedly heterogeneous enhancement (Figure 4). The disappearance of a normal high signal in the posterior pituitary is the most characteristic imaging finding on MRI. Sometimes, only the pituitary stalk thickened can observed. Suprasellar germinomas are very sensitive to radiotherapy and the tumor can be significantly reduced or even disappear in follow up with imaging after irradiation.

**Figure 5:**
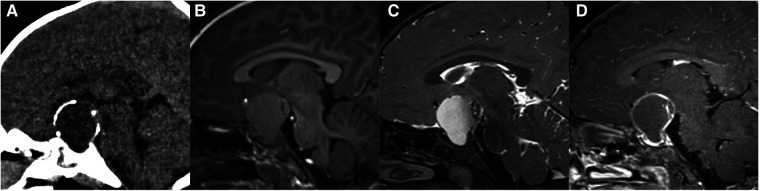
29-month-old girl with craniopharyngioma: sagittal unenhanced CT (**A**), T1WI (**B**), T2WI (**C**), and post-contrast T1WI (**D**) show a cystic mass with eggshell calcification in the sellar and suprasellar region.

**Figure 6:**
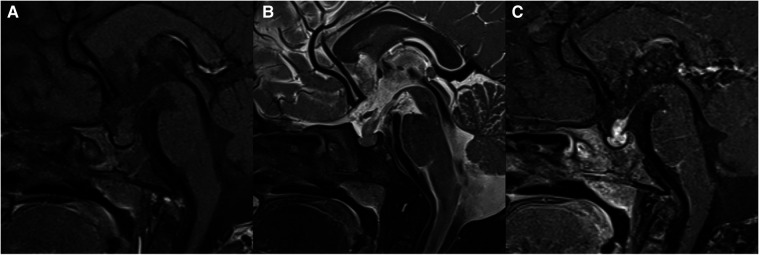
31-month-old boy with langerhans cell histiocytosis: sagittal T1WI (**A**), T2WI (**B**), and post-contrast T1WI (**C**) show thickened pituitary stalk with marked enhancement.

Differential diagnosis: (1) Craniopharyngioma: The distribution is bimodal with a first period occurrence between 5 and 15 years old and a second peak at around 50 years of age (26). Most of the patients are clinically diagnosed with visual field defects and intracranial hypertension. There is a lobulated, mixed solid/cystic mass commonly with eggshell calcification in the sellar/suprasellar region on CT images (Figure 5). MRI signals can be variable depending on tumor contents. The solid part is significantly enhanced. The normal high signal in the posterior pituitary can be seen. (2) Hypothalamic glioma: This originates from the hypothalamus and tends to grow along the optic pathway (27). Enlargement of the hypothalamus or optic chiasm is a typical imaging characteristic. The enhancement is usually not obvious, and cystic components or calcification are uncommon. The normal high signal in the posterior pituitary can be seen. (3) Langerhans cell histiocytosis: This is a common systemic disorder in children. Clinical presentations include exophthalmos, diabetes insipidus, and skull defects (28). The characteristic imaging findings on MRI are an isointense nodule in hypothalamus on T1WI, thickened pituitary stalk to a diameter greater than 3 mm, and marked enhancement (Figure 6). The normal high signal of the posterior pituitary disappears (4). Pituitary adenoma: Pituitary adenoma in children is very rare (29). MRI manifestations include an enlarged sella turcica and abnormal morphology and signal of the pituitary, often known as a “snowman” or “8” morphology. The high signal of posterior pituitary usually exists but is displaced or absent.

### Basal ganglia region

Germinomas in the basal ganglia region are relatively rare with a strong male predominance (25). The most common manifestation is slow progressive hemiplegia, with involuntary movement, cognitive decline, language disorder, and personality change (30).

**Figure 7:**
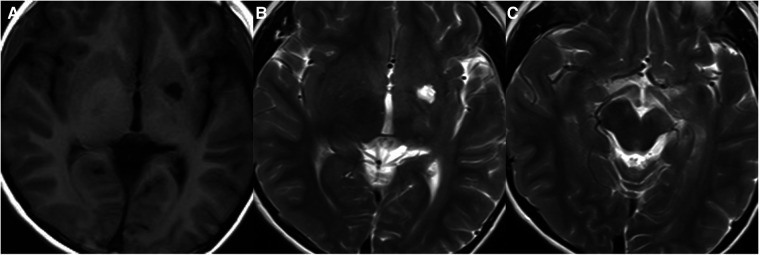
10-year-old boy with a basal ganglia germinoma: axial T1WI (**A**) and T2WI (**B,C**) show cystic change and ipsilateral atrophy of the cerebral peduncle.

In the early stage, germinomas in the basal ganglia region can show slight hyperdensity on CT, and slightly hyperintensity on T2WI with small cystic changes. Calcification can be seen on CT. Mild or no space-occupying effect is often observed. Hypointensity on T2* GRE/SWI and Wallerian degeneration of corticospinal tract, which is defined as ipsilateral atrophy of the cerebral hemisphere or cerebral peduncle, have been emphasized as reliable signs of basal ganglia germinoma (11, 30, 31) (Figure 7). Hypointensity on SWI is very important in identifying early stages of germinomas. On the contrast-enhanced scan, germinomas usually show no enhancement or slight patchy enhancement. It has been reported that ^11^C-methionine-positron emission tomography (MET-PET) may be helpful for the early diagnosis of basal ganglia germinomas when conventional MRI cannot diagnose them, especially for tumor contouring (32). Li et al. reported on 33 basal ganglia GCTs and found that, compared with NGGCTs, germinomas showed more cystic changes and inhomogeneous enhancement (30).

**Figure 8:**
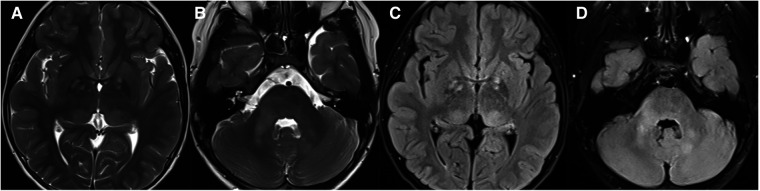
11-year-old boy with neurofibromatosis type 1: axial T2WI (**A** and **B**) and FLAIR (**C,D**) show hyperintensity in the bilateral basal ganglia, thalamus, brainstem, and cerebellum on T2WI/FLAIR.

Differential diagnosis: (1) Ischemic stroke in basal ganglia in adolescents: Early germinomas in the basal ganglia are often misdiagnosed as ischemic infarction. Ischemic stroke usually shows no hypointensity on SWI, and stenosis of the ipsilateral middle cerebral artery can be detected on MR angiography (33). (2) Unidentified neurofibromatosis objects (UNO): These are also known as focal areas of signal intensity (FASI) or unidentified bright objects (UBO). UNO show hyperintensity on T2WI/FLAIR and isointensity to hyperintensity on T1WI with no mass effect and enhancement, commonly identified in the basal ganglia (often the globus pallidus), thalamus, brainstem (pons), cerebellum, and subcortical white matter in children with neurofibromatosis type 1 (NF1) (Figure 8) (33).

Clinical and radiological characteristics of germinomas in different locations are shown in Tables 1, 2. We also summarized the differences in the typical clinical and imaging features of different types of intracranial midline tumors in Table 3.

**Table 1:** Clinical characteristics of germinomas in different locations.

	Proportion	Sex	Clinical symptoms
Pineal region	50%	Male > Female	Obstructive hydrocephalus, the Parinaud syndrome and endocrine disorders
Suprasellar region	20–30%	Female > Male	Diabetes insipidus, visual acuity decline, visual field defect and hypopituitarism
Basal ganglia region	5–10%	Male predominance	slowly progressive hemiplegia

**Table 2:** Imaging features of germinomas in different locations.

	Calcification	Boundary	Enhancement	Typical features
Pineal region	“Engulfed” calcification	Clear	Homogeneous	Bithalamic extension, thick peritumoral edema, isointense or slightly hyperintense on T2* GRE/SWI
Suprasellar region	Without calcification	Ill-defined	Heterogeneous	The normal high signal in the posterior pituitary disappears, thickened pituitary stalk
Basal ganglia region	With calcification	Clear or ill-defined	No or slight patchy	Wallerian degeneration, mild or no space-occupying effect, hypointense on T2* GRE/SWI

**Table 3:** Typical clinical and imaging features of different types of intracranial midline tumors.

	Location	Age	Typical imaging features
Germ cell tumor	Pineal, suprasellar region	10–20 y	“Engulfed” calcification, with restricted diffusion, the normal high signal in the posterior pituitary disappears, thickened pituitary stalk, spread along CSF, “bifocal germinoma”
Pineoblastoma	Pineal region	Infancy and childhood	Peripheral or “exploded” calcification, heterogeneous, with restricted diffusion and variable enhancement, invade adjacent structures and spread along CSF, hydrocephalus
Pineocytoma	Pineal region	Middle-aged adulthood	Clear boundary, peripheral calcification, without restricted diffusion, relatively homogeneous
Craniopharyngioma	Sellar, suprasellar region	5–15 y, around 50 years of age	Mixed solid/cystic mass with eggshell calcification
Hypothalamic glioma	Suprasellar region	Childhood	Enlargement of the hypothalamus or optic chiasm
Langerhans cell histiocytosis	Suprasellar region	Childhood	Isointense nodule in hypothalamus, thickened pituitary stalk
Pituitary adenoma	Sellar, suprasellar region	Puberty and adult	A “snowman” or “8” morphology

CSF, cerebrospinal fluid.

### Other rare regions

Rarely, germinomas may also occur in the ventricular system, cerebral hemisphere, cerebellum, brain stem, and corpus callosum (34–36). Clinical manifestations depend on the anatomical site of origin. Cystic changes are commonly observed. Since germinomas in these regions are extremely rare and present with nonspecific clinical symptoms and various imaging manifestations, preoperative diagnosis is extremely difficult.

### NGGCTs

NGGCTs are rare heterogeneous tumors that usually occur in the pineal region (24). They have a peak incidence in adolescents, slightly before the overall peak of germinoma, and with a male predominance (37). In addition, NGGCTs have a higher relapse risk and poorer outcomes. Since iGCTs have great differences in treatment and prognosis, preoperative imaging diagnosis and tumor markers are significant in guiding treatment.

**Figure 9:**
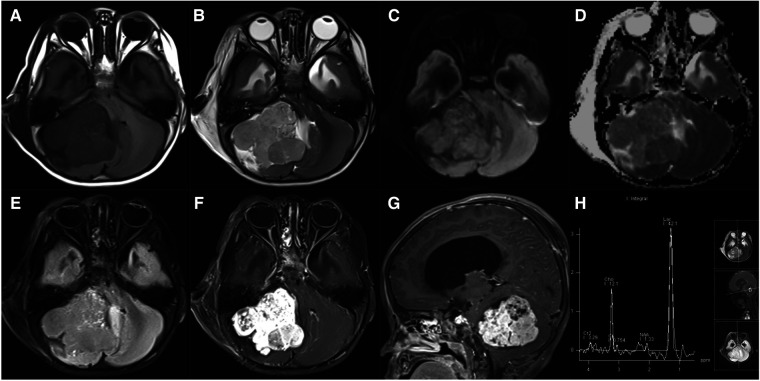
22-month-old boy with a cerebellar yolk sac tumor: axial T1WI (**A**), T2WI (**B**), DWI (**C**), ADC maps (**D**), FLAIR (**E**), and post-contrast T1WI (**F**), sagittal post-contrast T1WI (**G**), and MRS (**H**) show an irregular, heterogeneous mass with marked enhancement, diffusion restriction, and decreased NAA peak.

The typical imaging appearances of mature teratoma are heterogeneous masses with fat, calcification, cystic, and solid components. Except for mature teratoma, imaging features may be similar to iGCTs on conventional MRI/CT, while NGGCTs are relatively variable and heterogeneous, and aggressive findings may be present (Figure 9). One study showed NGGCTs were significantly larger than germinomas, the presence of intratumoral T1 hyperintense foci and heterogeneous moderate/marked enhancement were significantly more common in NGGCTs than in germinomas, and mean ADC values were significantly higher in NGGCTs than in germinomas (4). A previous study reported that solid portions of NGGCTs were prominently hypointense on T2* GRE/SWI due to hemorrhage commonly observed in NGGCTs (11). The arterial spin labeling (ASL) imaging revealed higher relative tumor blood flow within NGGCTs compared with germinomas (38). Therefore, the multimodal MRI application is useful for classifying subtypes of iGCTs (Table 4).

**Table 4:** Different MRI features between germinomas and NGGCTs.

	Shape	T1WI/C+	DWI	T2* GRE/SWI	ASL/ PWI	MRS
Germinomas	Clear or ill-defined	Hypointense to isointense/homogeneous contrast enhancement	Reduced ADC values	Hypointense to slightly hyperintense	Lower relative cerebral blood flow and time-to-peak values	Elevated lipid peaks and taurine peaks
NGGCTs	Significantly larger than germinomas	Intratumoral hyperintense foci /heterogeneous contrast enhancement	Significantly higher ADC values than germinomas	Prominent hypointensity	Higher relative tumor blood flow than germinomas	Elevated lipid peaks

MRI, magnetic resonance imaging; NGGCTs, non-germinomatous germ cell tumors; DWI, diffusion weighted imaging; T1WI, T1 weighted imaging; C+, contrast enhancement; ADC, apparent diffusion coefficient; T2* GRE/SWI, T2* gradient echo/susceptibility weighted imaging; ASL, arterial spin labeling; PWI, perfusion weighted imaging; MRS, magnetic resonance spectroscopy.

### Tumor marker detection

Routine examination of the tumor markers is highly recommended for patients suspending iGCTs and is especially helpful in diagnosing NGGCTs. The serum/cerebrospinal fluid (CSF) levels of beta-human chorionic gonadotropin (β-HCG) and alpha-fetoprotein (AFP) play an important role in aiding the diagnosis process. Tumor markers should be detected both in serum and CSF if possible, because it has been proven that the content of β-HCG in CSF is significantly higher than in serum, while the content of AFP in serum is slightly higher than in CSF (37). According to the standards of the international society of pediatric oncology (SIOP) and children's oncology group (COG), NGGCTs can be defined clinically by the elevation of AFP level (AFP >10 mg/L) in serum or CSF, which includes yolk sac tumors, immature teratoma, and embryonic carcinoma (24). The AFP concentration in a pure yolk sac tumor is typically greater than 500 mg/L. And the diagnosis of choriocarcinoma and embryonal carcinoma can be considered if there are raised β-HCG levels in serum or CSF (β-HCG >50 IU/L) (39). The concentration of β-HCG in a pure choriocarcinoma is typically greater than 1,000 IU/L. CD30, CK(AE1/AE3) and POU5F1 (OCT3/4) are expressed in embryonal carcinoma (40). Germinomas with a syncytiotrophoblastic component secrete β-HCG, which may lead to the elevation of β-HCG level (41, 42). In addition, most germinomas highly express placental alkaline phosphatase (PLAP), c-kit (CD117), and POU5F1 (OCT3/4) (24, 43). Differences in tumor markers in serum and CSF of iGCTs are shown in Table 5 (44–46). It is important to note that even if there were no changes in tumor marker levels, iGCTs cannot be excluded.

**Table 5:** Classifications of GCTs according to serum/CSF tumor markers.

	AFP	β-HCG	PLAP	c-kit
Germinoma	−	+/−	+++	+
Choriocarcinoma	−	+++	+	−
Yolk sac tumor	+++	−	+	−
Embryonal carcinoma	+/−	+/−	+	−
Mature teratoma	−	−	−	−
Immature teratoma	+/−	−	+/−	+/−
Mixed GCT	+/−	+/−	+/−	+/−

Symbols indicate the degree of elevation of the marker level, from lowest (−) to highest (+++).

GCT, germ cell tumor; CSF, cerebrospinal fluid; AFP, alpha-fetoprotein; β-HCG, beta-human chorionic gonadotropin; PLAP, placental alkaline phosphatase; c-kit, CD117.

### The significance of differential diagnosis between germinomas and NGGCTs for management

The distinction between germinomas, NGGCTs, and other midline intracranial tumors is very important because the management methods, such as surgery, radiation therapy, and chemotherapy, differ for different tumor types. Currently, since germinoma is highly sensitive to radiotherapy and the harm of surgical complications outweighs the benefit, the surgery is not recommended for germinoma and is most commonly treated with craniospinal irradiation alone, which yields a cure rate of more than 90% (24). NGGCTs usually require an aggressive treatment such as complete resection or a combination of surgery and other therapy.

Therefore, the differential diagnosis of germinomas and NGGCTs through MRI and the serum/CSF tumor markers is essential. We have already mentioned the differences between them in Tables 3–5. At present, a biopsy is generally not suggested if tumor markers in the serum/CSF and radiological appearances are typical of iGCTs. The MRI scan can also be used for follow-up of response to treatment.

## Conclusion

Taken together, T1WI, DWI, T2* GRE/SWI, ASL, PWI, and MRS can provide useful additional information to aid in the differential diagnosis of iGCTs subtypes. The serum/CSF tumor marker detection is also very helpful for differential diagnosis. Future research may introduce artificial intelligence and radiomics methods to accurately analyze images of iGCTs, combined with gender, clinical symptoms, and tumor markers, which is more useful in predicting tumor subtypes and guiding clinical decision-making.
